# “It’s more than a ride” veteran perceptions of peer specialist qualities and activities that were most valuable for post-incarceration reentry: a qualitative analysis

**DOI:** 10.1186/s40352-024-00303-7

**Published:** 2025-01-10

**Authors:** Eric Richardson, Kimberlee Flike, Justeen Hyde, Beth Ann Petrakis, D. Keith McInnes, Bo Kim

**Affiliations:** 1 Center for Health Optimization and Implementation Research, VA Boston Healthcare System, Boston, USA; 2 Susan and Alan Solomont School of Nursing, Zuckerberg College of Health Sciences, University of Massachusetts Lowell, Lowell, MA USA; 3 Center for Health Optimization and Implementation Research, VA Bedford Healthcare System, 200 Springs Road, MS 152, Bldg. 70, Rm 285, Bedford, Bedford, MA 01730 USA; 4 General Internal Medicine, Boston University School of Medicine, Boston, MA USA; 5 Department of Health Law Policy and Management, Boston University School of Public Health, Boston, MA USA; 6 Department of Psychiatry, Harvard Medical School, Boston, MA USA; 7 Justice 4 Housing, 23 Bradston Street, Boston, MA USA

## Abstract

**Background:**

Reentry veterans experience many barriers to achieving physical and psychological well-being. While peer specialists can provide important support to veterans as they readjust to life post-incarceration, their specific activities and qualities most valued by veterans are not well known. The Post-Incarceration Engagement (PIE) intervention, coordinated with VA’s Health Care for Reentry Veterans (HCRV) program, links reentry veterans with a peer specialist who provides connection to services and social-emotional support during the reentry process. We conducted a qualitative examination of veterans’ perceptions regarding the key qualities and activities of peer specialists that were most valued during their reentry process.

**Methods:**

We interviewed 25 veterans engaged in PIE about their experiences working with a PIE peer specialist. We conducted a thematic analysis. Two project team members independently coded interviews and identified emergent themes that were refined with input from other members.

**Results:**

Veterans found the peer specialist’s physical and emotional availability, shared lived experience, and connection to resources to be invaluable for successful reentry post-incarceration. Veterans emphasized how important it was that the peer was consistently available and provided social, emotional, and logistical support. Secondly, veterans found it valuable to work with another veteran familiar with the VA system and to be able to share lived experiences. It provided an instant connection with the peer specialist. Finally, the personalized connections to VA and community resources equipped the reentry veterans with the essential resources to ensure continued success post-incarceration.

**Conclusion:**

Reentry veterans identified several key qualities and activities of the peer specialist that were vital to their reentry success. Our results may be used to inform other interventions aimed at improving the lives of reentry veterans along with other reentry populations.

**Supplementary Information:**

The online version contains supplementary material available at 10.1186/s40352-024-00303-7.

## Introduction

Annually, an estimated 15,000 veterans need connection to vital social support to help them successfully reintegrate back into their communities after incarceration (Bronson et al., 2015). Often hindered by heightened risk factors (e.g., homelessness, substance use disorder), these “reentry veterans” encounter a complex array of tasks (e.g., applying for or reclaiming benefits, finding employment/housing, legal requirements) to achieve well-being and successful reintegration (Finlay et al., 2017; Humphreys et al., 2018; Joudrey et al., 2019; Tsai et al., 2013). In contrast to traditional emphases on risk and recidivism, emerging trends describe successful reintegration not only as avoiding recidivism, but also achieving overall psychological and physical well-being referred to as desistance (Blue-Howells et al., 2013; Finlay et al., 2017, 2019; Fox, 2022). Desistance is a process where a shift in a sense of identity results in someone reducing criminal behaviors while moving towards a greater emphasis on positive identities and overall well-being. Social support is crucial for veterans to engage in this process of achieving well-being through modeling and reinforcing healthy behaviors. As they navigate the reentry process, reentry veterans need to re-learn social norms, and build a network of support necessary for overall well-being and reintegrating into communities (Chouhy et al., 2020; Mowen & Boman, 2019). Yet, reentry veterans are often socially isolated with few positive role models to help reintegrate to the community upon release from incarceration (Finlay et al., 2017). Thus, connecting reentry veterans with positive social support is critical for establishing desistance.

Peer specialists are ideal for providing the needed positive social support to enhance reentry Veterans’ transition after incarceration towards desistance, and early literature on effectiveness is promising (Adams & Lincoln, 2020; Bellamy et al., 2019). Peer specialists leverage their own lived experiences with recovery, incarceration, or living with a mental health disorder, to deliver services and support for individuals (Adams & Lincoln, 2020; Chinman et al., 2014). Because they’re able to be available for routine activities, they provide many services that can encompass navigation of healthcare, education, role modeling, and emotional support (Chinman et al., 2014, 2021). While, no systematic reviews on peer specialist reentry interventions promoting desistance have been published, there are several studies showing promising results on the effects of peer specialist interventions to reduce recidivism (i.e., reduce criminal behaviors) and increase connection to needed resources during the early reentry transition period (Bagnall et al., 2015; Bellamy et al., 2019; Hyde et al., 2022a, b). For example, in a 2019 pilot study of 50 men being released from prison, those working with a peer specialist post-release had a 21% chance of re-incarceration; this doubled (43%) for those not working with a peer specialist (Bellamy et al., 2019).

**Fig. 1:**
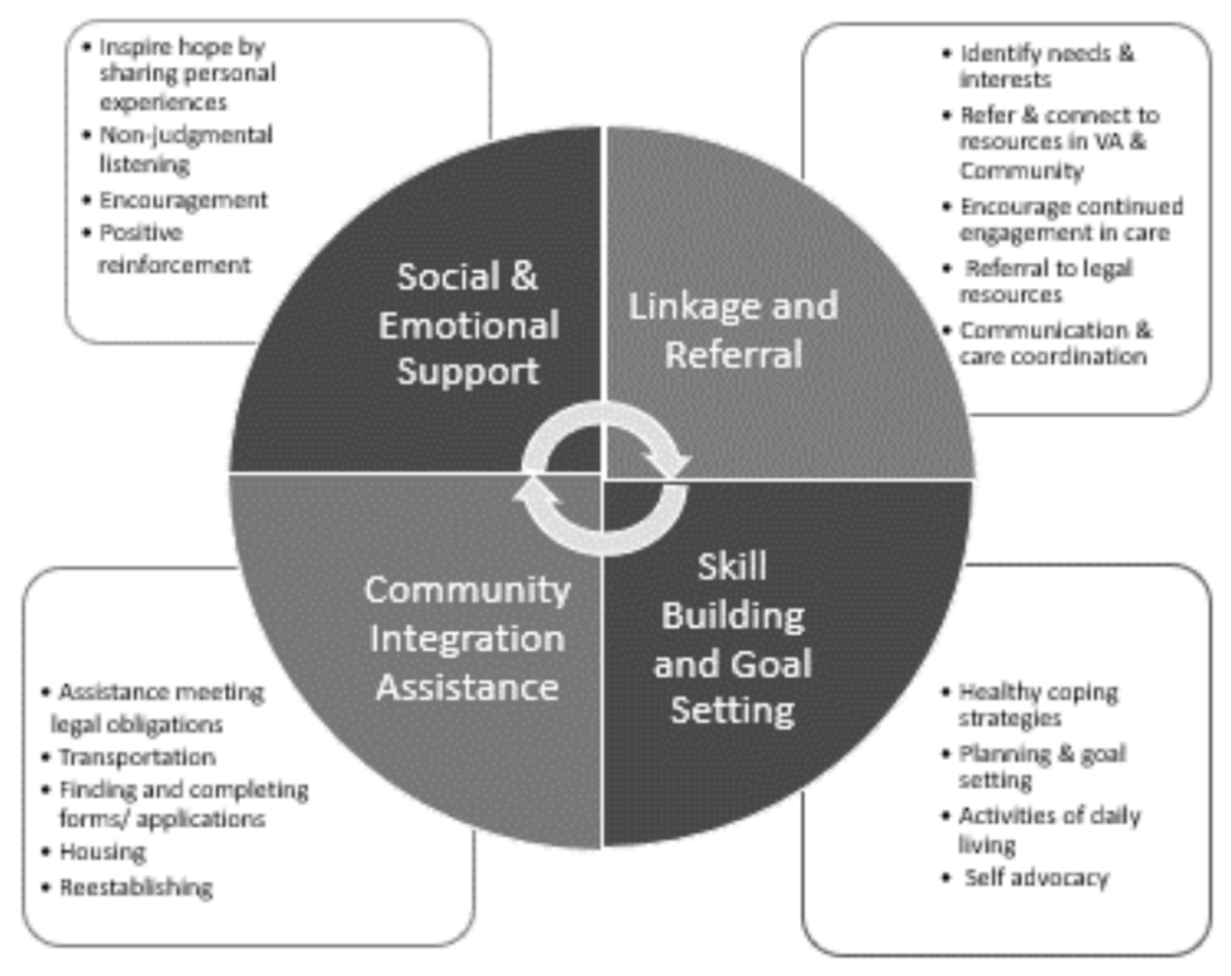
Core activities of the Post-Incarceration Engagement (PIE) Peer Specialist Program. **Legend**: The PIE program was designed to address four domains to assist reentry Veterans as they navigated their return to the community. These domains included (1) social and emotional support, (2) linkage and referral to VA and community resources, (3) community integration assistance and (4) skill building and goal setting. Examples of peer activities associated with each domain are given in the box attached to the domain

Another example, the Post-Incarceration Engagement (PIE) intervention, leverages the Department of Veteran Affairs (VA) increasing use of peer specialists to support veterans reintegrating into the community early after release from incarceration (Hyde et al., 2022a, b; Johnson et al., 2022; Kim et al., 2019; Simmons et al., 2017). The PIE intervention connects veterans just released from prison with a VA peer specialist to help facilitate successful reentry by connecting them to VA and community services. Peer specialists are paired with reentry veterans for a period of approximately six months post-release. The PIE intervention was developed through formative evaluations (Hyde et al., 2022a, b}, and builds on the VA’s Whole Health program, which is defined as “approach to healthcare that empowers and equips people to take charge of their health and well-being and live their life to the fullest.”(Bokhour et al., 2020, 2022) Thus, the goal of the PIE intervention is to enhance existing VA reentry services (i.e., Healthcare for Reentry Veterans, HCRV) by incorporating a Whole Health approach focused on what matters most to the veteran. PIE provides veterans with social and emotional support, linkage and referral to needed services, skill building and goal setting, and community reintegration assistance (Fig. 1). Social support is central to the underpinnings of PIE. Peers engage in guided discussions to learn what the veteran wants their post-incarceration life to look like, tailors action steps and goals, and then supports the reentry veteran. Once paired with a peer specialist, veterans receive different types of support, tailored to their individual interests and needs (Simmons et al., 2017). The tailored support provided by the peer improved critical reentry outcomes that can increase well-being such as connection to healthcare and remaining in the community. Based on a pilot trial, veterans participating in PIE were more likely to engage in substance use treatment (86% vs. 19% in control group, *p* < 0.001) and mental health services (93% vs. 64%, *p* < 0.003), and only 7% were arrested during the study period (Hyde et al., 2022a, b).

While the effectiveness of peer specialists supporting reentry veterans is promising, more research is needed to know what makes these relationships effective in fostering desistance among reentry veterans (Gidugu et al., 2015). Particularly, exploring the key qualities and specific activities of peer specialists that reentry veterans found most impactful may provide insight into promoting successful reentry and increasing the likelihood of desistance. To this end, we conducted interviews with reentry veterans who received peer support from the PIE intervention. Our analysis sought to identify veterans’ perceptions of (1) key activities that most impacted and/or facilitated veterans’ reentry experiences, and (2) qualities of the peer specialist that impacted the peer specialist-veteran relationship. We share our findings below, followed by a discussion of how they can inform the future implementation of forensic peer specialist (i.e., peers with knowledge of the criminal justice system) interventions.

## Methods

### Design

As part of a larger summative evaluation of the PIE intervention pilot trial, we conducted and analyzed semi-structured interview data collected from veterans who participated in PIE for 6 months or longer. The interviews assessed veterans’ perceptions and experiences of working with a peer specialist during the 6-month intervention period. The project was reviewed by the VA Bedford Healthcare System Institutional Review Board and was designated as a quality improvement project.

### Participant recruitment

Between July 2018 and January 2020, we recruited and completed semi-structured interviews with 25 veterans that had participated for at least 6-months in an implementation pilot of the PIE intervention in a Northeastern state. As part of the summative evaluation of the PIE intervention, members of the evaluation team contacted veterans to invite them to participate in an interview to share their experiences and feedback of their participation in the PIE intervention. Veterans that agreed to participate were then scheduled for an interview with a member of the evaluation team.

### Eligibility to participate in PIE

To be eligible to participate in PIE, veterans had to (1) be incarcerated and scheduled for release within six months or recently released (< 6 months) back into community settings, and (2) eligible for VA healthcare services. HCRV’s outreach specialists identified eligible veterans while still incarcerated or shortly after release and introduced them to PIE peers. Additional details regarding the procedures used to recruit veterans to the PIE intervention are described in a previous publication (Simmons et al., 2017).

### Data collection

A semi-structured interview guide was developed to systematically evaluate the veterans’ perceptions and experiences of participating in PIE program as part of a larger evaluation of the implementation and effectiveness of PIE. The interview guide consisted of 7 primary questions and additional associated questions. Probing follow-up questions were used to elicit additional information (e.g., “Can you tell more about that?”). Questions asked veterans to describe incarceration experiences, experiences navigating reentry, qualities of the peer specialist, activities the peer performed, the impact of working with a peer, positive/negative experiences over the previous 6 months, and recommendations to improve the PIE program. Example questions include: “When the opportunity to work with a peer (NAME or NAME) was presented, what did you think it would be like?; What role do you think your peer mentor (NAME or NAME) has played in your reentry experience?” Full interview guide can be found the supplemental materials.

Participants were asked to sign a consent for permission to record the interview and all participants included in this analysis freely consented to this. Two experienced interviewers (BAP, VY) conducted the interviews in person. Each interview took approximately 30 min (average of 31 min) to complete and at the end participants were given a $20 gift card to thank them. We audio recorded the interviews using a VA approved recording device and following the interview, they were saved in a secure folder. Recordings were professionally transcribed verbatim (by VA’s Centralized Transcription Services Program (CTSP)) using a denaturalized approach for analysis. The transcription service performed an independent quality check prior to sending the completed transcripts. Interview transcripts were uploaded into NVivo 12.0 (QSR International) for analysis.

### Data analysis

We followed an established method for conducting thematic analysis in healthcare research that outlines four steps: (1) familiarization with the data through generation of codes, (2) identifying themes from the developed codes, (3) combine and/or further divide themes into categories, and (4) conceptualize a model that interrelates the themes. (Chapman et al., 2015)

#### Step 1: familiarization with the data through generation of codes

Qualitative researchers (JH, BAP, ER, KF, BK) developed a codebook inductively. Specifically, two researchers (JH, BAP) jointly reviewed one transcript and then each read two additional transcripts. Using this sample of 5 interviews they met to develop and revise a preliminary codebook with definitions. Then ER, KF and BK randomly selected a subsample of 20% of the transcripts as a trial for the drafted codebook. Based on these selected transcripts, the codebook was refined by BK, ER, and KF. Using the refined codebook, two researchers (ER, KF) independently coded each of these four transcripts and the remaining 21 transcripts, reconciling coding discrepancies through consensus and iteratively updating the codebook to refine the initial definitions and include any additional codes that were used to code the later transcripts. As needed, a third researcher (BK) was available to resolve discrepancies if consensus was not able to be reached. The third coder would weigh the decision process of the two primary reviewers then lead a discussion to reach unanimous agreement. Coding was conducted using NVivo 12 (QSR International, 2020).

#### Step 2: identifying themes from the codes

Using the NVivo qualitative software, we generated reports of each code. We focused our thematic analysis on the codes that described veterans’ descriptions of reentry experiences, activities of the peer, qualities of the peer, impact the peer had on reentry, and the recommendations to improve the PIE intervention. ER and KF independently read the reports of each code and identified relevant themes. The coding team (ER, KF, BK, BAP, JH) verified the themes.

#### Steps 3 and 4: combine/divide themes and conceptualize a model from the themes

Once we identified themes, the qualitative coding team met to compare themes and reached consensus on themes closely related to the data. We then reviewed and refined together with other project team members. Together with these project members, we conceptualized the three overarching themes related to peer support described in the results.

## Results

### Participant characteristics

Our sample (Table 1) included 25 veterans with an average age of 51 (Range 29–72). They were all male, mostly White (*n* = 19, 76%), and about half Divorced/Separated/Widowed (*n* = 12, 48%). A minority of the veterans (*n* = 9, 36%) served in a combat zone. A majority of the sample had multiple incarcerations (*n* = 19, 76%), almost half (*n* = 11, 44%) were charged with a violent crime, and the average length of the sentence was 53 months.

**Table 1:** Reentry veteran characteristics

	Sample *N* = 25
**Age Years (Range)**	51 (range 29–72)
**Race**	
White	76% (19)
Black/African American	12% (3)
American Indian or Native Alaskan	8% (2)
**Ethnicity**	
Hispanic	0% (0)
**Marital Status**	
Married/In a relationship	16% (4)
Single (Never Married)	32% (8)
Separated/Divorced/Widowed	48% (12)
**Served in combat zone**	36% (9)
**Offense**	
Violent Offense	44% (11)
Other	12% (3)
Property	24% (6)
Violation	16% (4)
Drug	4% (1)
**Prior Offences**	76% (19)
**Average Length of time incarcerated**	53 Months (9 Median)

### Overview of thematic findings

The veterans in this study described the difficulties they experienced during their initial reentry period. One veteran explained the initial challenges of working with someone new: *“People coming out of jail are usually untrustworthy of people they don’t know*, *so they are reluctant to ask for help when they need it.” (Veteran 10).* Another participant highlighted the deep roots of mistrust that can make it difficult to initially connect with someone:

*I keep my cards close to my vest. I always have*, *and I just explained to him [peer] that you*, *that the environment I’m coming from*, *that’s how you survive. And I’m still in that. I came from a battlefield. It really was a battlefield where I came from. (Veteran 14)*

The difficulties described by reentry veterans during the first few months of their lives post-incarceration provide a context for understanding the qualities and activities they reported valuing from their relationship with the peer specialists.

Based on our analysis, we identified three themes to describe what peer qualities and activities veterans found were most valuable during their involvement in the PIE intervention: (1) The peer was available: “Every time I called…he was there”, (2) The peer has shared experience: “Someone [who] has kind of walked in my shoes”, and (3) The peer connected them to resources: “He’s got all of the answers for most of the stuff”.

#### The peer was available: “Every time I called…He was there”

Veterans in our sample emphasized the peer’s availability as crucial in support of their reentry. The peer specialists were often described as “*always reliable*,” “*always there*,” and “*always available*.” The veterans indicated that “availability” included both physical and emotional availability of the peer specialist. Physical availability was demonstrated through the peer specialists going to the prisons and introducing themselves and the PIE program before the veterans’ release:

*I say that because I met [peer] while I was still in jail*, *so_ That was good*, *because I didn’t come out and then this guy was sprung on me. I got to meet him while I was still in there*, *and he told me what sort of things that he helps out with*, *what he does*, *what he doesn’t do. (Veteran 09)*

A commonly reported example of physical availability was transportation to important appointments post-release: “*They are very reliable*, *especially when it came to appointments with Veterans Court. They’d give me the ride there.” (Veteran 06).* Many veterans indicated transportation to medical appointments, court, and parole appointments, and running errands was difficult to obtain on their own. Peer specialists’ availability to help them get to these appointments was one of the most valuable aspects of the PIE program as it helped them meet a variety of legal, health, and logistical needs.

Although many veterans indicated how important the physical availability of the peer was, they also noted that the emotional availability of the peer specialist, demonstrated through traits such as being genuine and patient, was also very important to them. When recounting instances when the peer was available both physically and emotionally, a veteran stated, “*Oh*, *[peer specialist] was hand-in-hand with me. Like when I needed him*, *he was always there for transportation*, *when I needed an ear to talk to*, *when I needed advice.” (Veteran 12).* The veterans indicated they counted on the peer for transportation, but it was often more than just a ride. The peer used the time while driving the veterans to provide emotional support and foster a deeper connection: *“It wasn’t just a ride*, *but he was*, *he could identify with the alcoholic and the substance abuse type of things too.” (Veteran 05).* The combination of physical and emotional availability demonstrated through the acts of tangible support of the peer specialist had an important impact on the veterans’ reentry success and hope for a better future, as described by one veteran: *“You helped me out*, *and not just everything that you guys did for me*, *but just the fact that you’re there*, *that you exist. That’s a shining light. You know what I mean… It gives me hope for the future.” (Veteran 02).*

#### Peer has shared experience: “Someone [who] has kind of walked in my shoes”

Veterans in our sample found that the shared lived experience with peer specialists was instrumental in facilitating their reentry. In the case of the PIE program, the two peers in the pilot program had a shared experience of recovery, housing instability and being a veteran. The veterans indicated that these lived experiences that they shared with the peer specialists facilitated a meaningful connection early in the peer-veteran relationship:

*We connected right away*, *so he’ll be talking*, *and it will be like telling my story*, *so that’s why I could relate to him. And his personal stuff with his relationships with his wife or girlfriend or whatever*, *and it will be very similar to the experience that I’ve gone through. (Veteran 03)*

This recognition of a shared experience created a mutual understanding which underpinned the peer specialist-reentry veteran relationship as explained by one participant: *“But absolutely*, *having someone with*, *has kind of walked in my shoes*, *so to speak*, *that makes a big difference in navigating the complexity of the Veteran’s care*, *the Veteran’s resources. (Veteran 11)”.*

Participants indicated that there was a relatable, almost friend-like connection, that was helpful for providing guidance and direction:

*Because he was coming more from like a peer*, *a friend perspective. You know what I mean? It wasn’t like being over there*, *talking to staff. It was just different. They’re like firm and kind of stiff… It was just different with him. It was more like I was talking to a buddy of mine than it was like someone watching over me and providing me care. You know? (Veteran 07)*

Knowing the peers shared similar life experiences also made the participants feel less judged, which was particularly important because of the stigma they experienced from others: *“They were helpful because you felt like nobody was judging you. (Veteran 05)”.*

The shared experiences also allowed the veterans to see the peers as a role model for what is possible:

*You need someone there who is a pillar in the community*, *who is looking not only to help you but lead by example. You know what I mean? You look at a guy like [name] and [name]. These guys are good guys. They’ve done a lot of things in their life. They’ve always been*, *they’ve always been*, *you know what I mean? They’re just good upstanding pillars of the community that have been an example themselves. (Veteran 02)*

Participants were receptive to this positive role-modeling provided by the peers because the peers’ personal struggles followed by a successful recovery showed them what was possible in their own lives. One veteran described how the shared connections with the peer over prior substance use helped him understand how to avoid future pitfalls: *“Like*, *they gave*, *what I love about the way that they did was they took their experiences and helped me live through that. Like*, *I learn best through others’ experiences and their lessons they learned.” (Veteran 06).*

#### The peer connected them to resources: “He’s got all of the answers…”

When first released from jail or prison, veterans were often disoriented because they did not have the resources or knowledge to successfully start the reentry process. One veteran summarized the experience: *“I didn’t know nothing. You’ve got to understand that. I knew absolutely nothing. I knew no programs. I knew nothing of what existed. I came out. I was just in a fog. (Veteran 02)”.* Another echoed these sentiments and elaborated how the peer’s ability to help them navigate the VA helped him move forward in his reentry: *“And he helped me navigate through the various parts of the VA*, *which can be difficult too*, *because when you first get out*, *that first month is horrendous. It’s like you don’t have any idea what you’re doing.” (Veteran 01).*

Many of the veterans in our sample regarded the connections to VA and community resources that peers provided as a vital foundation for success during their reentry process. They reported that the peers connected them to many available resources necessary for successful community reintegration including housing, healthcare, support groups, financial resources, benefits programs and employment opportunities. One veteran explained how this connection to resources was an important for the initial transition from prison to the community:

*They showed me the program*, *CWT (Compensated Work Therapy). I can work there. They showed me housing*, *how to go about doing the housing*, *the VASH (VA Supportive Housing)*, *what things to fill out. How to get the basic necessities. And the basic necessities for me*, *coming out of prison*, *is basically the foundation. (Veteran 02)*

Veterans valued the peer specialists’ deep knowledge of the available resources and their willingness to connect with other providers if they did not already have an answer to a problem:

*And he knows the system*, *this whole system of where to go and what to do and this and that. He’s got all the answers for most of the stuff. If you have questions for him*, *he’s going to know. And if he doesn’t know*, *he’s going to know which direction to point you in to find out. (Veteran 13)*

The peer specialists were able to combine this knowledge of resources with their knowledge of the personality of the veteran to direct them toward opportunities that would provide the best outcomes. For example, stable housing is considered an essential component in the reentry process, and one veteran described how the peer’s insight into housing options was helpful when deciding what housing programs would best meet his needs:

*He gave me*, *him and [name] gave me a lot of different leads for transitional housing. Basically*, *he kind of told me what he thought would suit my personality*, *where I should go based off of his feedback from our interaction together. So I took that advice into consideration. (Veteran 14)*

The veterans described how the peer was able to tailor the resource connections they provided by completing goal-setting worksheets adapted for the PIE intervention. These worksheets served as a person-centered approach to tailor the supports and activities around the goals and activities that mattered most to the Veterans, as described by one Veteran:

*I felt that I had a better connection with [peer specialist] going through the series of questions and kind of talking through what my goals*, *short-term*, *long-term*, *what my needs are*, *what my next steps are*, *where do I go from here kind of scenario. (Veteran 11)*

This reflective process encouraged veterans to think about what they really want for themselves and start planning for their future. Peers offered support developing goals and action steps, helped to problem solve around barriers, and celebrated successes. The veterans also noted how the worksheets allowed them to track their progress and have a sense of accomplishment: *“…I think that the worksheets that I did*, *they helped me see how I was doing*, *how I was progressing. I’m sure they would be helpful to other people also.” (Veteran 08).* The veterans also indicated how the peers’ knowledge of the resources built on the first theme of availability, where being both available and providing useful services enhanced the Veteran’s reentry experience. This was summed by one veteran: *“And by showing that you’re here to help and to actually be here to help are two huge things. That and like a welcome package. Welcome home type of thing.” (Veteran 01).*

## Discussion

Reentry veterans reported that the peers’ consistent availability, shared life experiences, and making connections to a wide range of resources were some of the most valuable aspects of the social support provided. Consistent with previous social support literature, the availability of peer specialists to establish an early connection based on shared experience helped veterans cope with the initial transition from incarceration to the community (Chapman et al., 2018; Chinman et al., 2014). Our findings extend previous work by describing concrete ways that peer specialists can not only support reentry veterans during their transition back into the community, but also help veterans cultivate the necessary skills for successful long-term community reintegration (Bellamy et al., 2019; McNeill, 2006; Mowen & Boman, 2019). Moreover, our findings outline core reintegration activities of peer specialists that can be used in peer specialist training and increase fidelity across multiple sites for optimal reach.

The veterans in our sample described qualities and activities performed by the peer specialist that aligned with the four core element of the PIE intervention (See Fig. 1). Consistent with these elements, reentry veterans recounted the ways the peer specialist connected them to housing, modeled life skills, and helped them develop and navigate relationships. As such, social and emotional support seems most meaningful in the context of a peer relationship that can help overcome the barriers to basic needs and services (e.g., housing, healthcare). Despite the support services (other than peers) available to help reentry veterans, previous research has found almost half of reentry veterans do not have contact with VA health care once released from incarceration (Blue-Howells et al., 2013; Finlay et al., 2017; Palframan et al., 2020). These connections, however, are essential and can be lifesaving. From a sample of veterans connected to substance use treatment through HCRV, those in treatment were 63% less likely to die from a drug overdose, compared with reentry veterans not in treatment (Blonigen et al., 2017). A previous evaluation of the quantitative outcomes of the PIE pilot indicated many veteran participants were connected to VA services and established stable housing during their 6 months in the program (Hyde et al., 2022a, b). Our qualitative results provide additional insight into how these connections to services were facilitated by the peer specialists’ in-depth knowledge of both the VA system and their personal connections with, and insight into, the individual needs of the veterans. Furthermore, the veterans noted the significant impact that the transportation and emotional support provided by the peer specialist had on their reentry experiences, which may have contributed to the increase in engagement in services seen in our quantitative results. Peer specialists tailor the support that they provide to each veteran to best meet the veteran’s unique needs, assisting the Veteran gain the needed information and facilitate appropriate connections to meet their goals. As such, future studies evaluating the long-term outcomes of veterans who receive peer support are warranted.

Additionally, activities described by the veterans in our sample align with the wider peer specialist literature such as the importance of peers’ close proximity, shared experience, and social support in contributing to desistance among veterans. (Maruna & Roy, 2007; Pettus-Davis et al., 2011). Reentry interventions commonly connect people being released from incarceration early in the transition process either before being released or shortly thereafter which has been identified as vital to establishing rapport (Sells et al., 2020). This is due to a high risk of recidivism in the first 90 days after leaving incarceration, particularly among those experiencing homelessness, substance use disorder, or mental health disorders (Finlay et al., 2019; Humphreys et al., 2018; Tsai et al., 2014). These challenges can be magnified if the reentry individuals are isolated from positive social connections, which may encourage a return to previously established criminal patterns (Choucy et al., 2020; Mowen & Boman, 2018; Mowen & Boman, 2019). Reentry veterans in PIE noted that this early connection helped build needed rapport and provide tangible support (such as transportation). In addition, the consistent availability of peer specialists maintained the needed proximity between the peer and the veteran to further develop a meaningful peer relationship and help the veterans move in a positive direction as they readjust to living in the community. As with other peer support interventions, the shared life experience fostered a trusting relationship, one that provides direction and hope (Gidugu et al., 2015). Similar to other participants in reentry programs, reentry veterans from our sample expressed that shared experience reduced the feelings of judgement and made them more receptive to advice and role modeling (Lenkens et al., 2023). One way this manifested in our sample was the shared experience of recovery between the peer specialist and reentry veteran. By interacting with someone who overcame addiction, reentry veterans gained a sense of hope, and also were open to guidance from the peer, e.g. on reducing some behaviors and increasing others.

Our findings extend the literature on social support highlighting the importance of tangible services, facilitation of key relationships, and even small acts performed by the peer specialists; all of which enables social and emotional stability that is necessary for successful reintegration (Chouhy et al., 2020; Kay, 2022; McNeill, 2006). In other literature, there is mention of providing tangible support to address needs that are vital to desisting from criminal behaviors and improving well-being (Kjellstrand et al., 2023). For example, connection to stable housing is vital for successful reentry, and veterans in our sample described the ways peers provided guidance in finding housing. The guidance provided can be vital to larger life skills such as increasing social networks or emotion regulation that will be valuable for future desistance. Transportation is another example; the simple act of getting a ride was vital to getting benefits and arriving at crucial appointments; simultaneously peers used the time during rides to help veterans with goal setting and to provide them emotional support. Tangible actions, like a ride to a probation appointment, served as a conduit to vital emotional support that builds desistance. As future directions in reentry support emphasize more holistic reintegration than the traditional risk-recidivism approach, research can examine how the tangible actions of peers, such as those identified through this work, serve instrumental roles (Maruna, 2004; Chouhy et al., 2020).

Finally, our findings outline core activities that can be used in the on-going multi-site expansion of the PIE intervention. By identifying the peer activities and qualities most valuable to reentry veterans, PIE can be more consistently implemented across the sites to focus on those activities/qualities and can also help other peer specialist interventions develop core activities that align with what veterans indicated were most impactful. While there were only 2 peers in out pilot of PIE, the qualities veterans highlighted were stated to be important in the success of the program. Thus, when implementing an intervention like PIE, the characteristics described are applicable across sites. For example, iterations of PIE need to consider flexible peer schedules to meet the needs of the veteran, train the peers on resources to connect the veterans, and consider hiring people with similar lived experiences to those the program will serve. These activities and qualities highlighted could be applicable to non-veterans as they return to the community after incarceration. This is worth further exploration as high rates of recidivism are persistent among many previously incarcerated populations (Hirschtritt & Binder, 2017). In addition, the results of this study may be beneficial to other peer support programs, particularly those that address substance use, as many of the veterans in our sample reported prior substance use history (Johnson et al., 2022; Jordan et al., 2022).

Several limitations need to be considered when interpreting the results. First, due to the small sample of Veterans in this study, our results may not be generalizable to other reentry populations. Among veterans incarcerated, however, our sample was largely representative of the nationwide demographics. The Bureau of Justice Statistic’s most recent survey, conducted in 2016, showed that 98% of veterans in state and federal prisons were men and that they were older and more likely to be White and serving longer sentences than incarcerated nonveterans. Our sample largely aligns with these characteristics including a majority with prior offenses and almost half were incarcerated for a violent crime (indicative of a longer sentence). Second, this was a pilot in one Northeastern state and may not be indicative of reentry experiences from other regions with differing rules and procedures. Third, the Veterans in our sample only worked with two peers. Other literature suggests that this is not uncommon among reentry programs, according to one review the average program enlists 2–3 peer specialists (Adams & Lincoln, 2021). The PIE program is currently being implemented at additional sites, thus there will be an opportunity to see whether the qualities and activities are shared with a more diverse group of peer specialists. Finally, the current analysis is a secondary data analysis. There is a chance that secondary data analysis can misinterpret original data or be less rigorous. To ensure rigor, the members of the data collection team were involved in the analysis and reviewed the coding. Additionally, the research questions were closely aligned with the intention of the pilot to ensure continuity (Ruggiano & Perry, 2019).

In summary, according to reentry veterans, peer specialists can provide important social and instrumental support through offering consistent availability, leveraging a shared experience, and facilitating connection to needed resources. Reentry veterans found the PIE peer support intervention invaluable for navigating systems, role modeling important skills, and fostering a sense of hope for the future. Notably, the Veterans noted the peer’s availability to provide transportation and navigate complicated health and social services as very useful forms of support. The veterans also emphasized that shared experiences were important to develop trust and provide a sense of hope for the future. In addition, providing transportation was a service veterans valued, and it allowed time for social and emotional support and goal setting. The veterans also trusted the peers to provide personalized recommendations for available VA and community resources, which may have contributed to their increased use of these programs. Programs planning to implement or expand peer services should consider these key points when developing their intervention. Future research can use these activities and qualities to train peer specialists and develop fidelity measures to expand the reach of peer support for reentry veterans. Moreover, the peer actions identified through this work can serve as the basis for future research that explicitly tests effectiveness of those peer actions in improving reentry support for both veterans and others leaving incarceration.

## Electronic supplementary material

Below is the link to the electronic supplementary material.

Supplementary Material 1

## Data Availability

The data generated during this study is not publicly available. All data were collected as part of a quality improvement initiative and not approved by the U.S. Department of Veteran Affairs to share.
