# Supplementary material for: “It’s more than a ride” veteran perceptions of peer specialist qualities and activities that were most valuable for post-incarceration reentry: a qualitative analysis
Source: Health Justice. 2025 Jan 10;13:2. doi: 10.1186/s40352-024-00303-7 (PMC11721156; doi:10.1186/s40352-024-00303-7)
Supplement: Supplementary file 1 — Supplementary Material 1 [file 40352_2024_303_MOESM1_ESM.docx]

**PIE Client 6 month Follow-Up Questionnaire**

**(v.06-26-2018)**

***INTRODUCTION TO OPEN-ENDED SECTION*: You may know that the peer support that you have received from [NAME/NAME Walton] is part of a pilot project that we have developed to support Veterans coming out of incarceration in STATE. We appreciate you agreeing to take part in these services and helping us to learn how it works for you. We would like to ask you just a few more questions that could help us improve the peer support services model as we roll it out in other states. As with the rest of the questions we asked, this is not an evaluation of how well [NAME/NAME^[[1]](#footnote-1)^] performed; instead we ask these questions so that we can improve the program so that it is meeting the needs of Veterans like you.**

**Before I begin, would it be OK with you if I record this part of the interview so that I can take notes later (rather than trying to capture your thoughts while we talk now)? If we ever write a report using quotes we would never use your name.**

1. **When the opportunity to work with a peer (NAME or NAME) was presented, what did you think it would be like?**
2. Why did you agree to work with a peer?
3. To what extent did it meet your expectations?
4. **What role do you think your peer mentor (NAME or NAME) has played in your re-entry experience?**
5. What have they done that has been really useful? (Possibly ask to identify the 2-3 most helpful things)
6. What more could they have done [or different types of things] to support you in getting re-established in a community setting?
7. ***Throughout your time working with the peer, you’ve been given different worksheets (“My priorities”, My Plan of Action”).   What did you think about the worksheets?    (In what ways were they helpful? Not helpful?)***

***For the next 2 questions please write down how things have been going, focusing on the last 3 months.***

1. **In general, how has it been going for you in the last three months? What are you most proud of? What has been most frustrating?**

|  |
| --- |

1. **What have you felt like you needed the most in the last 3 months? To what extent have you been able to meet these needs? (This could be concrete things like transportation or money, or social needs like advice, someone to talk to, etc.)**

|  |
| --- |

***In the last two questions we ask you to think about your future and to help us plan next steps with this reentry peer program.***

1. ***Recently you may have talked with your peer about your goals and action plans for the future.  How do you feel now about going off and pursuing those goals and actions on your own?***
2. **We are hoping to offer peer support services for Veterans leaving incarceration in other states beyond STATE. As we plan to do this, what recommendations can you offer that would help us improve this kind of peer support service? Probe:**
3. How the concept of having a peer is introduced (messaging and communication)
4. Activities/interactions around the first days of release
5. Action planning (i.e. worksheets or discussions about steps to take to meet your goals)
6. Connection to services and resources
7. Length of time engaging with the peer (which was about 6 month, for most Veterans)
8. Explaining and taking part in the evaluation
9. Others?

1. [↑](#footnote-ref-1)
